# The Effect of Submicron Polystyrene on the Electrokinetic Potential of Cell Membranes of Red Blood Cells and Platelets

**DOI:** 10.3390/membranes12040366

**Published:** 2022-03-26

**Authors:** Marcin Zając, Joanna Kotyńska, Mateusz Worobiczuk, Joanna Breczko, Monika Naumowicz

**Affiliations:** 1 Doctoral School of Exact and Natural Sciences, University of Bialystok, K. Ciolkowskiego 1K, 15-245 Bialystok, Poland; m.zajac@uwb.edu.pl; 2 Department of Physical Chemistry, Faculty of Chemistry, University of Bialystok, K. Ciolkowskiego 1K, 15-245 Bialystok, Poland; joannak@uwb.edu.pl (J.K.); mworobiczuk@gmail.com (M.W.); j.luszczyn@uwb.edu.pl (J.B.)

**Keywords:** polymers, erythrocytes, platelets, electrophoretic light scattering, dynamic light scattering, FTIR spectroscopy

## Abstract

In recent years, many scientists have studied the effects of polymer micro- and nanostructures on living organisms. As it turns out, plastic can be a component of the blood of livestock, eaten by humans around the globe. Thus, it seems important to investigate possible changes in the physicochemical parameters and morphology of the cell membranes of blood morphotic elements (red blood cells and platelets) under the influence of polymer particles. The article presents research in which cell membranes were exposed to plain polystyrene (PS) and amino-functionalized polystyrene (PS-NH_2_) of two different sizes. The polymers were characterized by infrared spectroscopy and dynamic light-scattering methods. To analyze possible changes caused by polymer exposure in the structure of the membranes, their zeta potentials were measured using the electrophoretic light-scattering technique. The concentration of the polymers, as well as the exposure time, were also taken into the consideration during the research. Based on the obtained results, we concluded that 100 and 200 nm PS, as well as 100 nm PS-NH_2_, internalize into the cells. On the contrary, 200 nm PS-NH_2_ particles attach to cell membranes. Our study clearly shows that particle size and surface chemistry determine the interaction with biological membranes.

## 1. Introduction

Nowadays, it is difficult to imagine functioning without polymers. Polymers are used in various industries, such as construction [1], electrical engineering [2], and medicine [3]. In recent years, more and more researchers from around the world have become interested in the characteristics of nano- and microplastic. Submicron polymer particles are defined as molecules of plastic smaller than 5 mm in diameter [4]. They are separated into two types: (1) Primary plastics, which enter the environment in their micro- and nanoscopic state, and (2) secondary plastics, which are the result of continuous environmental impacts on plastic litter and yield progressively smaller polymer fragments. Polystyrene (PS) is one of the most commonly used polymers in the production of plastic materials, together with polyethylene and polypropylene [5]. Its use is found mainly in food packaging, which, due to COVID-19, became a very busy branch of industry. Synthetic polymers have become a serious concern for the life and health safety of living organisms, as it is becoming clearer just how omnipresent plastic is in the ecosystem. Human activity is the main cause of contamination of the environment with polymers. Additionally, plastic production is continually increasing. It is estimated to reach 33 billion tons in 2050 [6]. The consequences of polymer particle debris for wildlife are becoming better and better documented.

Factors affecting the breakdown of polymers into smaller structures include UV radiation [7] or the influence of microorganisms [8,9,10]. The presence of polymers has already been evidenced in soil [11], freshwater [12], oceans [13], plants [14], animal organisms [15], and food products [16]. Mass production of plastic articles in society poses great risks, as these items should be properly disposed of or recycled. If these processes do not take place, polymers will certainly end up in the environment. Given plastic’s ubiquitous nature and small dimensions, its ingestion and subsequent impact on living organisms is a growing cause for concern. At cellular and molecular levels, alterations of immunological responses, neurotoxic effects, and the onset of genotoxicity have been observed in water-dwelling organisms exposed to polystyrene particles [17,18].

Domestic pigs (*Sus domestica*) are particularly useful in biomedical research for several reasons. This species is characterized by high fertility and occurs in many genotypes and phenotypes, which is important for the selection of appropriate genetic material for medicinal studies [19,20,21,22]. The usage of biological material from pigs raises less opposition in laboratory tests than tests on dogs or cats. *Sus domestica* is very important in medical sciences such as surgery [23,24] and pharmacy [25,26] because of correlations between their bodies and humans. Pigs are not only comparable in size, but also have analogies to humans in the structure of their digestive, nervous, circulatory, and urinary systems. These similarities in the anatomy and physiology of *Sus domestica* and *Homo sapiens* are one reason biological material from pigs can be used for reliable modeling of cellular processes of human bodies.

Soil is also believed to be a pathway for micropolymers to enter living organisms. As it was found out, plastic particles can enter plants grown in heavily polluted areas [27]. This could lead to the consumption of polymers while eating contaminated vegetables by humans or animals. Other studies have shown that digesting processes do not break down plastic, and synthetic polymers can be a component of manure [28]. Scientists discovered polymers in the muscle tissue of marine animals, such as turtles [29] or fish [30]. It is indicated that plastic particles can also cross the blood–brain barrier. The consequence of this is the accumulation of polymers in the microglia cells, worsening their ability to multiply and leading to their death [31]. This information sheds a different light on the effects of polymers on human cells, including cell membranes. 

Thus, food consumption can be a direct path of entry to the human body for micropolymers. Scientists believe that polymer molecules can move up the food chain thanks to their presence in the bloodstream of animals [32]. Thus, any type of meat could be a potential route for micro- and nanopolymers to enter the human system. This could have very serious consequences for health and wellness. Many tests have to be carried out to assess the toxicity and transport pathway of micropolymers from livestock, e.g., pigs, to the human body.

Experimental studies of the electrical properties of natural cell membranes can contribute to our knowledge of their properties, function, and structure. The zeta potential (otherwise known as the electrokinetic potential, *ζ*) of membranes is an extremely important electrical parameter that depends on the composition of the cytoplasmic membrane. This parameter defines the stability of a system and allows one to determine whether modifications of the natural system with polymers affect changes in the structure of the natural membrane. The zeta potential varies according to many parameters, such as temperature, pH, conductivity (ionic strength), and solvent (viscosity). Thus, small modifications to any of these parameters may have a significant influence on the zeta potential values [33]. One technique used to estimate zeta potential is electrophoretic light scattering (ELS), a technique based on dynamic light scattering (DLS), in which the shift in the frequency or oscillation phase of the laser beam depends on the mobility of particles/cells in an alternating electric field [34].

The purpose of the research presented in this article is to assess the influence of PS particles on the zeta potential of *Sus domestica* blood components—red blood cells (RBCs), also referred to as erythrocytes and platelets (thrombocytes). We chose polystyrene as it does not generate reactive oxygen species in the presence of cells, and membrane damage by oxidative stress is unlikely [35]. Two sizes of two types of polystyrene were used: 100 nm plain polystyrene (PS-100) and 200 nm plain polystyrene (PS-200), as well as 100 nm polystyrene with amino groups (PS-NH_2_-100) and 200 nm polystyrene with amino groups (PS-NH_2_-200). In addition, different particle concentrations and exposure times to the polymer were applied. Based on the knowledge of the authors, such a thorough analysis has never been performed before.

## 2. Materials and Methods

### 2.1. Materials

#### 2.1.1. Domestic Pig Blood

The blood was obtained from the Bost Meat Plant in Turosn Koscielna from adult pigs of the species *Sus domestica*. Animal blood was collected directly into sterile test tubes made of unbreakable plastic with buffered sodium citrate in a ratio of 1:9 to biological material. Those test tubes were delivered to the laboratory, where individual morphotic elements of blood were immediately isolated.

#### 2.1.2. Polymers

The following polymers were used to modify the biological membranes of the morphotic blood elements of *Sus domestica*: PS-100 and PS-200 (purchased from Sigma Aldrich, Saint Louis, MO, USA), PS-NH_2_-100 (purchased from Polysciences, Hirschberg an der Bergstrasse, Germany), and PS-NH_2_-200 (purchased from Bang Laboratories, Fishers, IN, USA). Each polymer was used at the concentrations of 0.002, 0.01, 0.1, and 0.5 mg/mL. The polymers were diluted to the appropriate concentration using 0.155 M sodium chloride, in which the blood was also suspended for physiological conditions. In preliminary experimental studies, the stability and durability of the tested polymers in the electrolyte solution were investigated using the DLS method. The tests were performed immediately after mixing morphotic blood elements with polymers and after 1 h and 3 h of exposure to plastic particles.

### 2.2. Methods

#### 2.2.1. Isolation of Erythrocytes from Blood

The blood samples (2 mL volume) were centrifuged at 900 rpm for 8 min at room temperature. The red blood cells fell to the bottom of the tube, while the thrombocyte-rich plasma was removed from the system and further isolation was performed. The erythrocytes were washed three times with a 0.155 M NaCl solution at 3000 rpm for 15 min to obtain a clean erythrocyte-rich system. Finally, the washed red blood cells were suspended in an electrolyte solution (0.155 M NaCl) to prepare them for measurement via electrophoretic light scattering.

#### 2.2.2. Isolation of Thrombocytes from Plasma

The plasma was centrifuged at 4000 rpm for 8 min. Thrombocytes remained at the bottom of the tube after centrifugation. The plasma was removed from the vial. The platelets were washed three times with 0.155 M sodium chloride, as were the erythrocytes. Finally, the thrombocytes were added to the electrolyte solution (0.155 M NaCl). This solution was then used during measurements performed by the ELS technique.

#### 2.2.3. Comparison of Different Polymers by FTIR Spectroscopy

The Fourier transform infrared (FTIR) measurements were performed on the Nicolet 6700 spectrometer (Thermo Scientific, Madison, WI, USA) in transmission mode using the KBr method. First, the stock solutions of the different PS microspheres (PS-100, PS-200, PS-NH_2_-100, and PS-NH_2_-200) were diluted with distilled water to a concentration of 0.1 mg/mL. Next, aqueous PS solutions were frozen with liquid nitrogen and lyophilized in a freeze dryer (Christ Alpha 1–2 LD plus with double–chamber, Osterode am Harz, Germany) for 24 h under 0.013 mbar pressure. The obtained powder samples were mixed and ground in an agate mortar with dry KBr at a mass ratio of 1:100. The powders pressed into KBr pellets were subjected to FTIR analysis. The FTIR spectra of different PS microspheres were collected at room temperature in the range 4000–400 cm^−1^ with a wavenumber resolution of 4 cm^−1^.

#### 2.2.4. Determination of Polymers Size

The DLS method was used to determine the durability and stability of polymer particles in 0.155 M sodium chloride at pH = 7.4 at 25 °C. The polymer particles suspended in the electrolyte were illuminated with a helium-neon (He–Ne) laser with a wavelength of 633 nm. Measurements were performed with a Zetasizer Nano ZS apparatus (Malvern Instruments, Malvern, UK). Using the Stokes–Einstein equation, the apparatus converted the particle velocity caused by Brownian motion into particle distribution. When determining the particle size, the Non-Invasive Back Scatter method was used, in which the detector is positioned at an angle of 173 degrees. The measured value is the hydrodynamic diameter. Size distributions by numbers were obtained. The resulting values were burdened with an error expressed as a standard deviation. All PS had a polydispersity index (called PDI, used to describe the variation in size) lower than 0.3. Additionally, analysis using the ELS technique was carried out that allowed for determining the zeta potential values of the tested systems.

#### 2.2.5. Determination of Zeta Potential

The zeta potential of both erythrocytes and thrombocytes was obtained by performing microelectrophoretic assessments on samples using the ELS technique. The experiment was performed as a pH function using a WTW InoLab pH 3310 laboratory meter (WTW, Weinheim, Germany). Blood components were suspended in a 0.155 M NaCl solution and titrated to the desired pH (range 3–12) with strong acid (HCl) and strong base (NaOH) solutions, prepared with sodium chloride to maintain the strength of the ionic solution constant. Polystyrene was added to NaCl to achieve the final concentrations of each polymer type: 0.002, 0.01, 0.1, and 0.5 mg/mL. The particles suspended in sodium chloride were exposed to an electric field during the measurements. The Zetasizer Nano ZS apparatus (Malvern, Great Britain) uses the Phase Analysis Light-Scattering technique to determine the electrophoretic mobility of the tested particles. Then, the electrophoretic mobility is converted into zeta potential. Experiments were conducted eight times with similar results obtained.

#### 2.2.6. Statistical Analysis

The data obtained in this study are expressed as mean ± SD. The data were analyzed by use of standard statistical analyses, namely one-way ANOVA with Scheffe’s F test for multiple comparisons to determine the significance between different groups. Values of *p* < 0.05 were considered significant.

## 3. Results and Discussion

An investigation into the characteristics of the polymers used in the research was carried out to analyze the occurrence of possible flaws in the commercial product that could affect the conducted analysis. The hydrodynamic size and stability of the submicron polystyrene particles were measured using the DLS method. FTIR spectroscopy was also utilized during the research for identity analysis.

To investigate the interactions between the cell membranes of morphotic blood components (erythrocytes and thrombocytes) and various polystyrene particles (PS-100, PS-200, PS-NH_2_-100, PS-NH_2_-200), several experimental studies were carried out using the ELS method. These interactions could potentially change the values of the electrokinetic potential of the systems studied. Both the influence of the polymer concentration and the exposure time of the cell membrane to the polymer were taken into the account during the experiment. Each of the measurements was performed as a function of the H^+^ ion concentration.

### 3.1. Characteristics of Polymers Used in the Study

**Figure 1:**
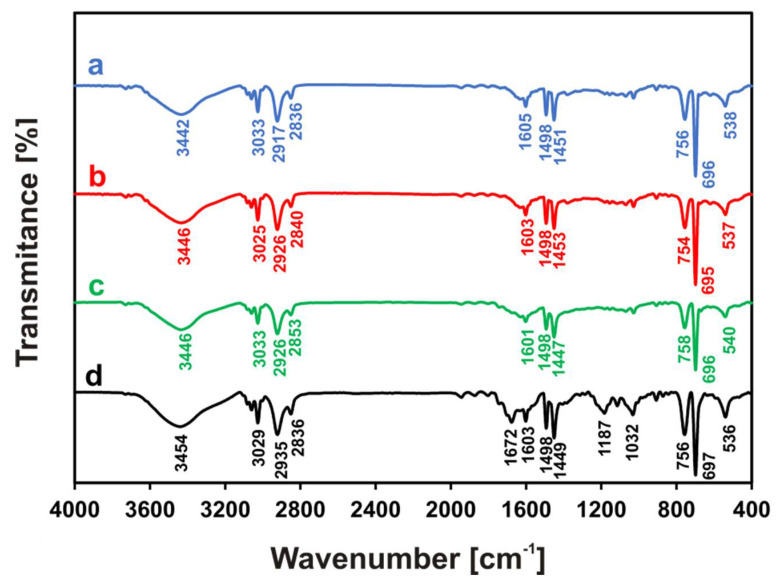
FTIR spectra of (**a**) PS–100, (**b**) PS–200, (**c**) PS–NH_2_–100, and (**d**) PS–NH_2_–200.

Structural studies of PS particles differing in size and/or the presence of amine groups on their surface were carried out using FTIR spectroscopy. Firstly, each of the analyzed polymers was suspended in water, and then the obtained sample was frozen and freeze-dried. The FTIR analysis of PS-100, PS-200, PS-NH_2_-100, and PS-NH_2_-200 is shown in Figure 1.

As expected, the recorded spectra of all samples showed bands confirming the structure of polystyrene [36,37,38]. The bands at approximately 3025–3033 cm^−1^ were attributed to the C–H stretching vibration of the benzene ring, while the signals at approximately 2917–2935 and 2836–2853 were assigned to the aliphatic –CH_2_– stretching vibration. Further proof of the presence of the benzene ring in the structure of the analyzed particles is the bands at 1605, 1498, and 1451 cm^−1^ (e.g., values for PS-100, Figure 1a), corresponding to the stretching vibrations of the aromatic C=C bonds. The bands at approximately 756 and 696 cm^−1^ (e.g., values for PS-100, Figure 1a) were characteristic of the aromatic substitution pattern [36,37] and were assigned to the C–H out-of-plane bending vibration. A broad peak at approximately 3442–3454 cm^−1^, corresponding to the stretching vibrations of O–H, indicated the existence of hydroxyl groups likely coming from the water. All obtained spectra (Figure 1a–d) looked much the same, differing slightly in the position of the bands that are characteristic of polystyrene. Additionally, the size of the analyzed particles did not result in any significant structural differences (PS-100, PS-200, Figure 1a,b). However, in the case of the amino-functionalized PS particles (PS-NH_2_-100, PS-NH_2_-200, Figure 1c,d) additional bands were observed, thus confirming their surface modification. The signal observed in the PS-NH_2_-200 spectrum (Figure 1d) at 1672 cm^−1^ and the bands appearing at 1187 and 1032 cm^−1^ were assigned to the N-H bending vibrations characteristic of primary amines and the stretching vibrations of C-N bonds, respectively. The analogous peaks in the FTIR spectrum recorded for PS-NH_2_-100 (Figure 1c) exhibited a low intensity and are not clearly visible. The reason for this is likely the smaller size of the tested submicron particles and the proportionally smaller number of –NH_2_ groups on its surface.

**Figure 2:**
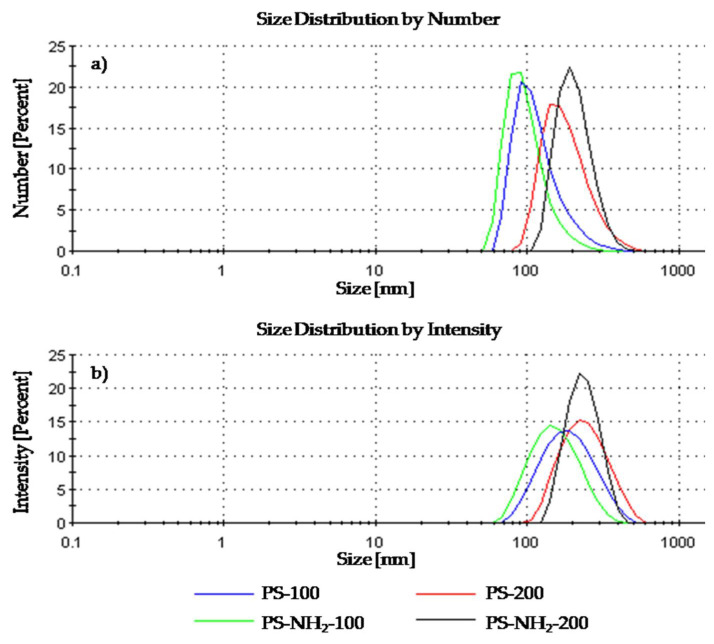
Polystyrene size distribution by (**a**) number, (**b**) intensity.

Parameters (size and zeta potential) characterizing the polymers were measured in 0.155 M sodium chloride at pH = 7.4 with the Zetasizer Nano ZS apparatus. The results obtained using the DLS technique are shown in Figure 2.

The plots of the polymer size distribution by number and intensity indicated the formation of monomodal fractions. The data obtained from dependences of the particles’ diameter by number range from 110 to 207 nm, and regarding intensity, range from 160 nm to 249 nm. The values registered for PS-100 and PS-NH_2_-100 differ slightly from the ones confirmed by the companies, while for PS-200 and PS-NH_2_-200, the measurements are consistent with the information provided by the producer.

The presence of other fractions, above 3000 nm in diameter, should not be ruled out. All size data obtained, including the standard deviation (SD) and polydispersity index (PDI) values, are collated in Table 1.

Summaries for the values of zeta potential are also compiled in Table 1, measured for each polymer using the ELS technique. The lowest zeta potential values were recorded for plain polystyrene particles with a size of 200 nm (−46.10 ± 2.11 mV). The remaining polymers (PS-100, PS-NH_2_-100, and PS-NH_2_-200) show *ζ* values ranging from −24.90 ± 1.20 mV to −31.00 ± 0.99 mV.

Generally, a large positive or negative value of the zeta potential (lower than −30 mV and higher than +30 mV) indicates physical stability due to the electrostatic repulsion of individual particles. On the other hand, a small value of the electrokinetic potential may cause the aggregation or flocculation of particles due to the van der Waals forces [39]. Based on data collected in Table 1, it can be concluded that all polymers are characterized by good stability.

### 3.2. The Effect of Polystyrene Polymers on the Zeta Potential of Morphotic Components of Pig Blood

#### 3.2.1. The Effect of Polystyrene Polymers Concentration

The ELS technique was used to provide insight into the possible changes in zeta potential values, due to treating erythrocytes and thrombocytes’ cell membranes with PS-100, PS-200, PS-NH_2_-100, or PS-NH_2_-200. In order to obtain the pH-dependent *ζ* value, the systems, suspended in a 0.155 M NaCl solution, were titrated to the appropriate pH with concentrated NaOH or HCl. 

**Figure 3:**
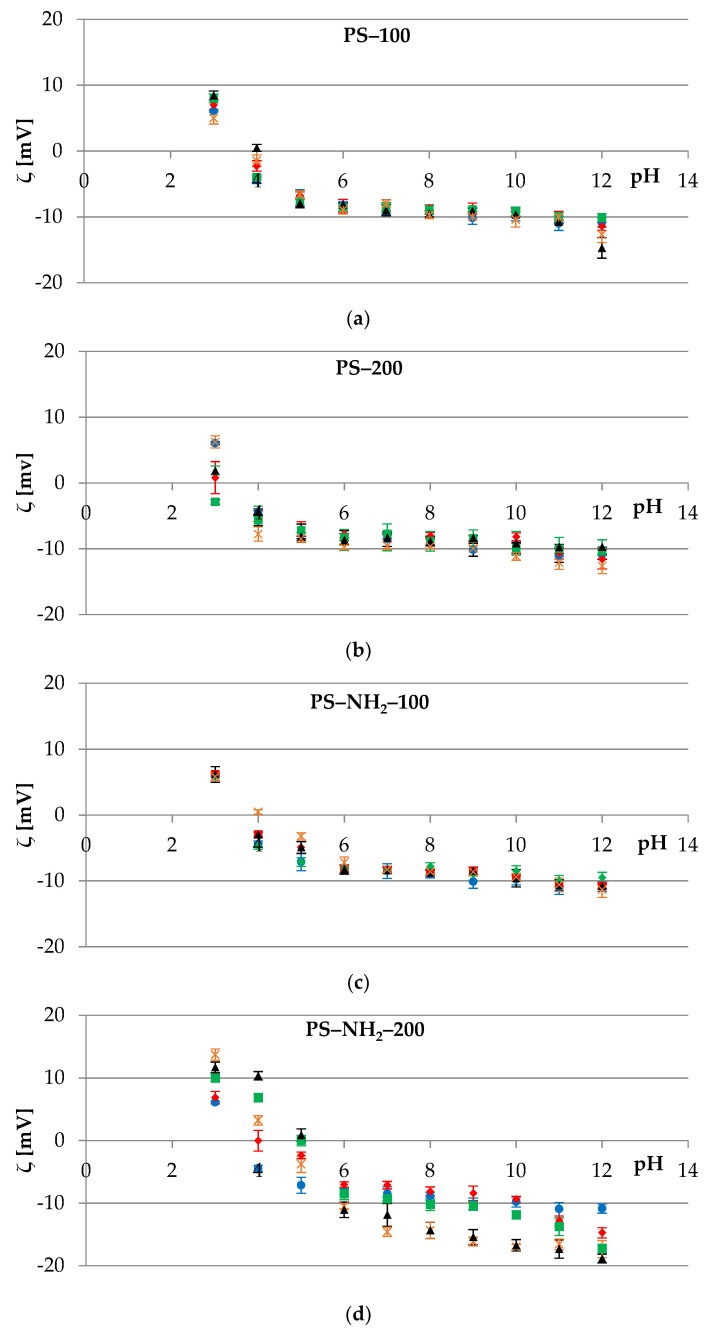
The zeta potential of the erythrocytes’ cell membranes as a function of the pH of the electrolyte solution. Membranes untreated (●) or treated with 0.002 (♦), 0.01 (■), 0.1 (▲), and 0.5 (×) mg/mL of various types of polystyrene polymer: (**a**) PS–100, (**b**) PS–200, (**c**) PS–NH_2_–100, and (**d**) PS–NH_2_–200.

Figure 3 shows representative plots of the electrokinetic potential vs. pH obtained for cell membranes of red blood cells modified with submicron polystyrene particles differing in size and/or the presence of amino groups on their surface. As it can be observed, an increase in the positive value of the zeta potential was observed alongside a decrease in the pH value. Conversely, as the pH increased, the negative values of *ζ* increased until they reached a plateau.

Upon analyzing data depicted in Figure 3, it can be noted that mixing red blood cells with three types of polystyrene particles (PS-100, PS-200, and PS-NH_2_-100, Figure 3a–c) did not cause statistically significant changes in *ζ* values compared to the control sample, which was pure erythrocytes suspended in 0.155 M sodium chloride. These changes were not noticeable in almost the entire range of polymer concentrations, with exceptions at extreme pH values where destruction of the membrane structure occurred. This was seen for membranes measured in the electrolyte solution containing 0.01 mg/mL PS-200 at pH~3 or 0.1 mg/mL PS-100 at pH~12. The lack of zeta potential changes allowed us to conclude that PS-100, PS-200, and PS-NH_2_-100 were internalized by the cell. The entry mechanisms for nanoparticles into cells are still not yet understood. None of the endocytic pathways, all of which involve vesicle formation in an actin-mediated process, are likely to account for nanoparticle translocation. The translocation of particles may also occur via non-specific pathways, including diffusion, trans-membrane channels, electrostatic, hydration, van der Waals forces, or steric interactions [40]. Moreover, PS particles within cells are not membrane-bound and hence have direct access to intracellular proteins, organelles, and DNA, which may greatly enhance their toxic potential [41]. 

In contrast, statistically significant changes in the *ζ* values of the erythrocyte cell membranes were observed after their treatment with PS-NH_2_-200 in the entire pH range studied, as shown in Figure 3d and Table 2.

Erythrocytes, the same as most biological surfaces, can be characterized by the negative charge occurring on their surface. This attribute is due to the presence of sialic acid located on the glycoproteins on the exposed parts of the cell [42]. As it is rather troublesome to measure the true surface charge atop cells, zeta potential is used interchangeably, having been deduced from electrophoretic mobility measurements. This parameter is a crucial characteristic of biological membranes, as it is bound to stabilize red blood cells dispersed in an electrolyte solution as it repels erythrocytes from other types of cells and, especially, themselves. This way, adhesion between RBCs and interactions with the endothelium is regulated [43]. Durocher et al. [44] proved mature cells have less sialic acid and less surface charge than those still maturing. These changes were theorized to occur during the senescence. 

Due to electrostatic interactions between negatively charged erythrocytes and ions of positive charge present in the structure of polystyrene, a disruption of the natural process occurs. Furthermore, while treating red blood cells with PS-NH_2_-200, the zeta potential values change as a result of generating additional surface charge atop the cells. Based on the data presented in Figure 3d, it can be presumed that PS-NH_2_-200 is attached to the RBC’s membrane. As a consequence of this phenomenon, the zeta potential values of erythrocytes treated with this type of plastic are increased in the range of pH 2–7. However, *ζ* tends to decrease in a solution with a pH above 7 as OH^−^ ions start to react with the amine groups of the PS-NH_2_-200 particles. 

The process of adhesion to the membranes and internalization into the cells of many particles is influenced by their physical properties, including size, shape, solubility, surface composition, and surface charge [45,46]. This leads to polymers of identical structures but distinct diameters causing different, sometimes contrary, effects on the natural surfaces, as size is a determinant of the mechanism of interaction between plastic and biological membranes [47].

Functionalized polystyrene particles become adhered to erythrocytes due to distinct mechanisms, hydrogen bonding, hydrophobic interactions, and specific van der Waals forces, to name a few [48,49]. As has already been described, PS with carboxyl groups present on their surface ranging in size from 100 nm to 1.1 μm and non- and amine-functionalized PS 200 nm in diameter become easily attached to erythrocyte surfaces [48,49]. It was also proven that increasing the number of particles present per cell led to more particles attaching to the red blood cells’ membranes. Erythrocytes’ morphology did not change due to the adhesion, and the investigated process was observed to be non-specific to any area of the cell surfaces [48,49]. However, polystyrene submicron particles of no charge, as well as of positive and negative charges, were found inside the red blood cells (while they were smaller than 0.1 μm in diameter) in addition to being present on the cell surface (while ranging from 0.2 to 1 μm in size) [40]. What is more, PS 78 nm and 200 nm in diameter were found within erythrocytes without inhabiting RBC’s membranes [41].

**Figure 4:**
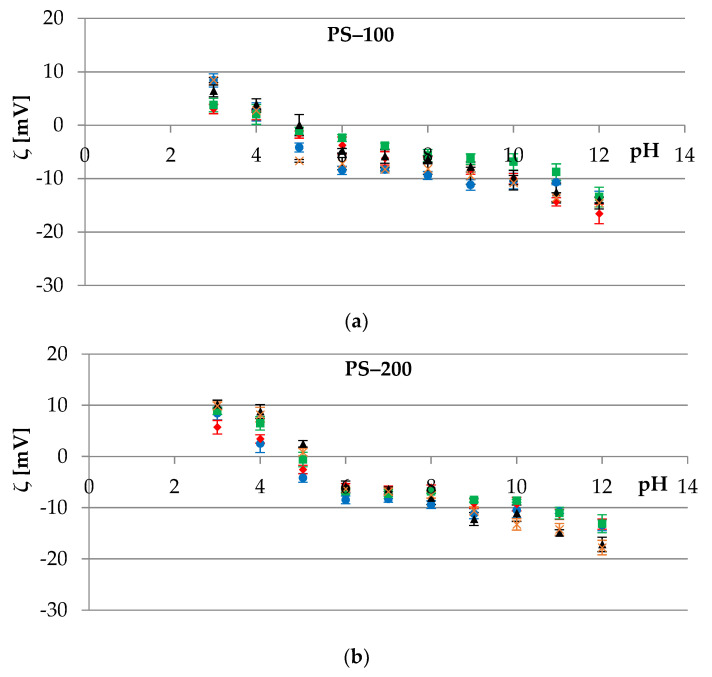

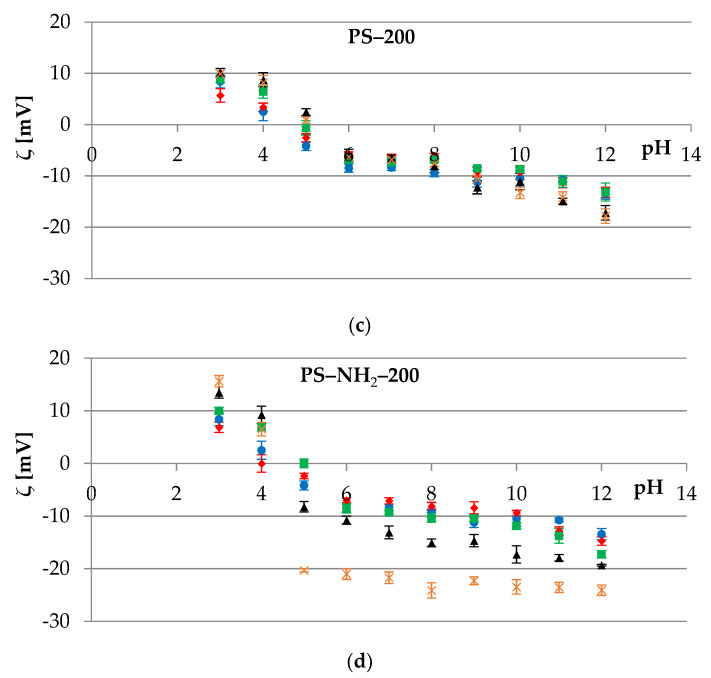
The zeta potential of the platelets’ cell membranes as a function of the pH of the electrolyte solution. Membranes untreated (●) or treated with 0.002 (♦), 0.01 (■), 0.1 (▲), and 0.5 (×) mg/mL of various types of polystyrene polymer: (**a**) PS–100, (**b**) PS–200, (**c**) PS–NH_2_–100, and (**d**) PS–NH_2_–200.

Platelets, similarly to erythrocytes, possess membrane glycoproteins that play an important role during two crucial processes: Adhesion to the subendothelial matrix and platelet–platelet cohesion, or aggregation [50]. These glycoproteins in their structure contain carboxyl groups that provide a negative charge to the surface of thrombocytes. The results for experimental studies of platelets’ cell membranes, treated and untreated with polystyrene polymers, are shown in Figure 4. Slight *ζ* changes were observed for membranes in contact with PS-100, PS-200, and PS-NH_2_-100 (Figure 4a–c), and the most visible were noted at extreme pH values, e.g., at pH~12 when 0.1 or 0.5 mg/mL PS-200 was used (Figure 4b). It was theorized that the changes result from the destruction of the cell membrane structure in an excessively alkaline environment. 

Statistically significant changes, as in the case of erythrocytes, were observed for the thrombocytes’ cell membranes treated with PS-NH_2_-200 (Figure 4d), and the most visible in the entire pH range were noted for polymer concentrations of 0.1 and 0.5 mg/mL. At an acidic pH (pH~3 and pH~4), the *ζ* reached higher positive values, while in the remaining pH range (pH 5 to 12), the zeta potential increased towards negative values. The changes that occurred in the parameter with the participation of lower concentrations of polystyrene (0.002 and 0.01 mg/mL) were insignificant.

The data of the zeta potential of thrombocytes during PS-NH_2_-200 treatment as a function of pH is compiled in Table 3. 

There is much evidence in the literature that suggests the possibility of interactions between submicron polymer particles and elements/components of the circulatory system—blood and blood vessels. The existence of such interactions would explain the proatherothrombic effect observed in a number of models. Smyth et al. [51] conducted research investigating the ability of non-functionalized and functionalized polystyrene particles of different sizes (50 and 100 nm) to cause thrombocytes’ aggregation in vitro and in vivo. Their results confirmed that the process of aggregation was influenced by the physical properties of PS. Tested particles caused GPIIb/IIIa-mediated aggregation of thrombocytes to a certain degree, varying due to the size and presence of surface groups. PS-NH_2_ of 50 nm in diameter acted in an enhanced agonist-induced aggregation by linking adjacent platelets, many of which were not even activated. Nemmar et al. [52] incubated thrombocytes with 60 nm polystyrene particles with different surface chemistries and found that the potency of causing aggregation was highest when using aminated particles, followed by carboxylated ones. Unmodified polystyrene was observed to induce aggregation to the lowest degree. Although these findings were to be generally confirmed by McGuinnes et al. [53], a difference in causing aggregation between PS particles of amino and carboxyl groups present on their surfaces was not to be found.

Given the data acquired using the ELS technique presented in Figure 3 and Figure 4 and Table 2 and Table 3, it can be recognized that significant changes of zeta potential were observed when treating erythrocytes and thrombocytes’ membranes using PS-NH_2_-200. The exposition of cells’ surfaces to 200 nm amine-functionalized polystyrene caused the electrokinetic potential to shift towards more negative values. As it was reported in the scientific literature, the larger the PS-NH_2_ was, the higher *ζ* tended to increase [54], which was confirmed in Section 3.2.1. We hypothesized that the interaction between erythrocyte and thrombocyte membranes and the PS-NH_2_-200 underlie the increased membrane perturbation. McGuinnes et al. [53] demonstrated that amino-functionalized PS appear to act via an unexplained mechanism that results in the display of anionic phospholipids on the outer surface of platelets and erythrocyte membranes and suggested that unusual protein adsorption patterns might lead to membrane perturbation-mediated aggregation.

#### 3.2.2. The Effect of the Exposure Time

Subsequent studies were focused on the measurement of the zeta potential of red blood cell and platelet membranes as a function of pH depending on the exposure time of the membranes to different polymers. Experimental research was performed immediately after suspending the blood components in a 0.155 M NaCl solution containing the polymer at a concentration of 0.1 mg/mL (at which changes in relation to the control sample were observed) and after 1 h and 3 h exposure of erythrocytes and thrombocytes to the polymer.

**Figure 5:**
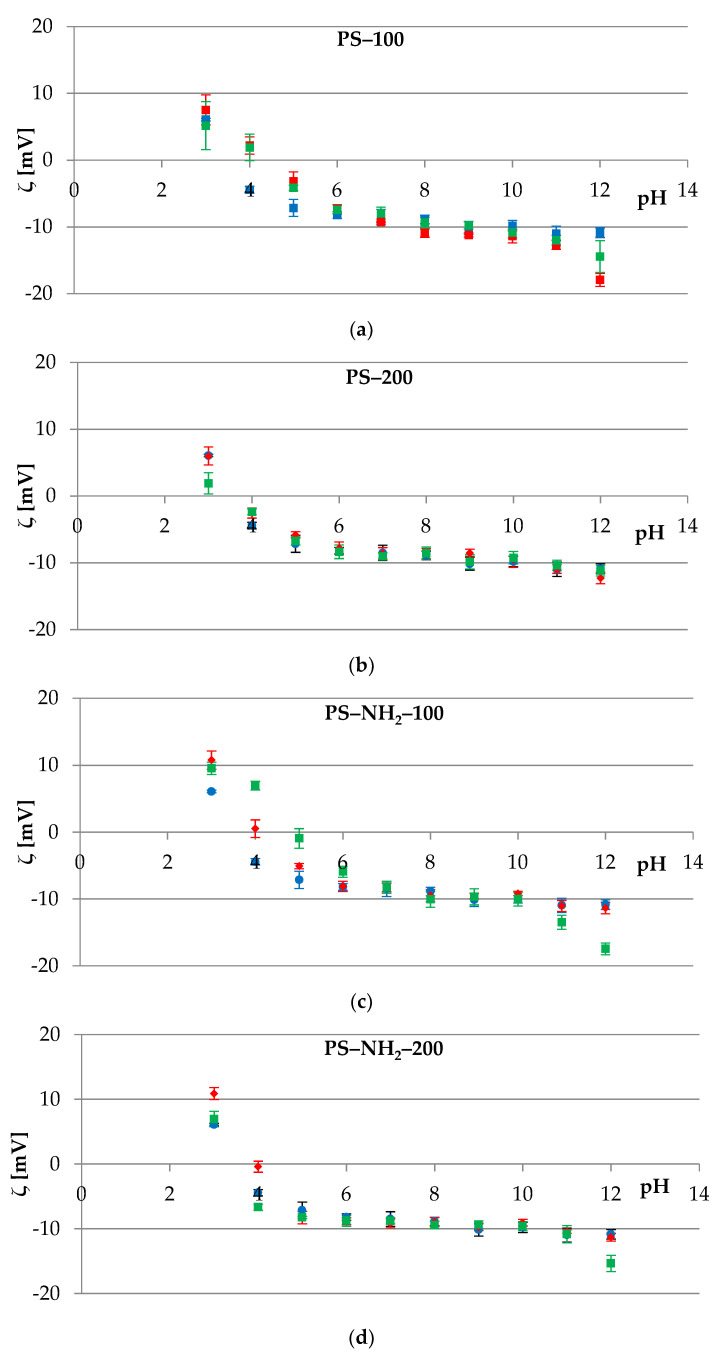
Zeta potential of the erythrocytes’ cell membranes as a function of the pH of the electrolyte solution. The parameter was measured immediately (●), after 1 h (♦), and 3 h (■) of exposing the sample to the polymer: (**a**) PS–100, (**b**) PS–200, (**c**) PS–NH_2_–100, and (**d**) PS–NH_2_–200.

The dependence of the zeta potential as a function of pH for RBCs is shown in Figure 5. Statistically significant changes were observed at the lowest and the highest pH values. Moreover, changes were observed in the case of (1) PS-100 at pH 4-5 (Figure 5a), (2) PS-NH_2_-100 in a pH range from 4 to 6 (Figure 5c), and (3) PS-NH_2_-200 at pH = 4 (Figure 5d). 

All particles used in this study were associated with erythrocytes—attached to or internalized by cells, depending on the size and surface chemistry. From the data shown in Figure 5, it can be seen that both processes occurred quickly and efficiently within one hour of the cells and particles coming into contact. Any coincidental discrepancies between the zeta values obtained for different durations of cell exposure to the polymer are likely due to the possibility of particles detaching from erythrocytes, as a result of shear forces, cell–cell interactions, and cell–vessel wall interactions [49].

**Figure 6:**
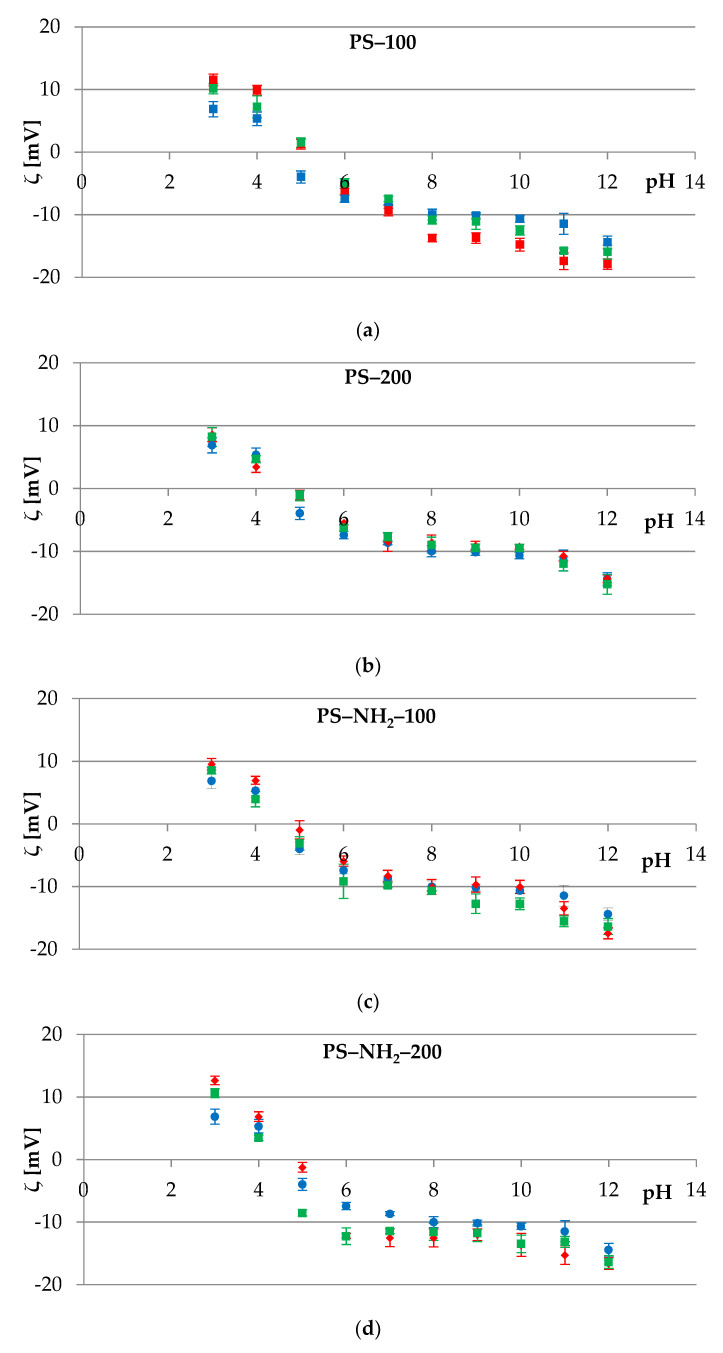
Zeta potential of the platelets’ cell membranes as a function of the pH of the electrolyte solution. The parameter was measured immediately (●), after 1 h (♦), and 3 h (■) of exposing the sample to the polymer: (**a**) PS–100, (**b**) PS–200, (**c**) PS–NH_2_–100, and (**d**) PS–NH_2_–200.

In the case of studies on thrombocyte membranes (Figure 6), significant changes in the *ζ* potential were observed while exposing these cells to PS-100 and PS-NH_2_-200.

From the data depicted in Figure 6, it can be noted that PS-200 and PS-NH_2_-100 quickly and efficiently attached to platelet membranes within an hour. For PS-100 and PS-NH_2_-200 particles, the association of polystyrene with cells was slower. This can be seen in the obtained dependencies, in which the zeta potential values measured after 3 h of thrombocyte exposure to the polymer are more similar to those measured directly than to those determined after 1 h of cell exposure. In the subject literature, studies have been presented in which internalization was noticeable as early as 1 min after incubation [46], and in which, as the duration of cell exposure to polystyrene increased, a greater probability of polystyrene association with cells was reported [37].

## 4. Conclusions

To summarize, there is a need to understand the influence of submicron polystyrene particle properties, such as size and chemical composition, on the various endpoints of particle toxicity. In the presented research, we investigated whether polystyrene particles influence the electrokinetic potential of erythrocytes and thrombocytes. Two types of polymers were used, and each experiment was conducted several times. In addition, different particle sizes and concentrations, as well as exposure durations to the polymer, always provided the same results, i.e., the internalization of PS-100, PS-200, and PS-NH_2_-100 particles into the cells and the attachment of PS-NH_2_-200 particles to cell membranes. Our study clearly shows that the size and surface chemistry of PS particles determine the extent to which they affect the electrical properties of thrombocyte and erythrocyte membranes.

## Figures and Tables

**Table 1:** The parameters characterizing the polymers (0.155 M NaCl, pH = 7.4).

Polymer	Size by Number [nm]	SD	Size by Intensity [nm]	SD	PDI	Zeta Potential [mV]	SD
PS-100	119.90	47.04	197.40	76.24	0.257	−31.00	0.99
PS-200	185.70	67.86	249.40	86.00	0.205	−46.10	2.11
PS-NH_2_-100	110.10	34.01	160.70	58.50	0.231	−24.90	1.20
PS-NH_2_-200	207.90	54.82	238.40	57.04	0.055	−28.60	1.32

**Table 2:** The zeta potential of the erythrocytes’ cell membranes after exposure to PS-NH_2_-200 (C = 0.002, 0.01, 0.1, and 0.5 mg/mL).

pH	Control	0.002	0.01	0.1	0.5
3	6.07 ± 0.19	6.86 ± 0.98	9.97 ± 0.65 ^a,b^	11.68 ± 0.86 ^a,b,c^	13.72 ± 0.90 ^a,b,c,d^
4	−4.44 ± 0.50	−0.02 ± 1.65 ^a^	6.85 ± 0.67 ^a,b^	10.35 ± 0.67 ^a,b,c^	3.23 ± 0.75 ^a,b,c,d^
5	−7.15 ± 1.28	−2.37 ± 0.52 ^a^	0.00 ± 0.83 ^a,b^	0.86 ± 1.00 ^a,b^	−3.78 ± 1.31 ^a,c,d^
6	−8.20 ± 0.51	−7.03 ± 0.45	−8.53 ± 0.91	−11.06 ± 1.22 ^a,b,c^	−10.49 ± 0.39 ^a,b,c^
7	−8.51 ± 1.13	−7.12 ± 0.60	−9.31 ± 0.30	−11.88 ± 1.82 ^a,b,c^	−14.58 ± 0.75 ^a,b,c,d^
8	−8.89 ± 0.64	−8.15 ± 0.75	−10.24 ± 0.94	−14.34 ± 1.28 ^a,b,c^	−14.54 ± 1.32 ^a,b,c^
9	−10.15 ± 0.98	−8.43 ± 1.16	−10.43 ± 0.64	−15.42 ± 1.20 ^a,b,c^	−16.12 ± 0.71 ^a,b,c^
10	−9.81 ± 0.79	−9.41 ± 0.52	−11.88 ± 0.66 ^a,b^	−16.72 ± 0.90 ^a,b,c^	−16.96 ± 0.38 ^a,b,c^
11	−10.97 ± 1.08	−12.84 ± 0.80	−13.76 ± 1.41 ^a^	−17.34 ± 1.45 ^a,b,c^	−16.58 ± 0.86 ^a,b,c^
12	−10.86 ± 0.73	−14.74 ± 0.82 ^a^	−17.26 ± 0.70 ^a,b^	−18.84 ± 0.68 ^a,b^	−17.34 ± 1.36 ^a,b^

^a^ Statistically significant differences vs. control group, *p* < 0.05. ^b^ Statistically significant differences vs. modified erythrocyte membranes with PS-NH_2_-200 (C = 0.002 mg/mL), *p* < 0.05. ^c^ Statistically significant differences vs. modified erythrocyte membranes with PS-NH_2_-200 (C = 0.01 mg/mL), *p* < 0.05. ^d^ Statistically significant differences vs. modified erythrocyte membranes with PS-NH_2_-200 (C = 0.1 mg/mL), *p* < 0.05.

**Table 3:** The zeta potential of the platelets’ cell membranes after exposure to PS-NH_2_-200 (C = 0.002, 0.01, 0.1, and 0.5 mg/mL).

pH	Control	0.002	0.01	0.1	0.5
3	8.40 ± 0.58	6.86 ± 0.98	9.97 ± 0.65	13.48 ± 1.07 ^b^	15.28 ± 0.89 ^a,b,c^
4	2.49 ± 0.77	−0.02 ± 1.65	6.85 ± 0.67 ^a,b^	9.25 ± 1.62 ^a,b^	7.17 ± 0.93 ^a,b^
5	−4.19 ± 0.85	−2.37 ± 0.52	0.00 ± 0.83 ^a,b^	−8.24 ± 1.00 ^a,b,c^	−22.18 ± 1.82 ^a,b,c,d^
6	−8.48 ± 1.72	−7.03 ± 0.45 ^a^	−8.53 ± 0.91 ^b^	−10.78 ± 0.76 ^a,b,c^	−21.14 ± 0.95 ^a,b,c,d^
7	−8.35 ± 1.24	−7.12 ± 0.60	−9.31 ± 0.30 ^b^	−13.10 ± 1.21 ^a,b,c^	−22.48 ± 1.61 ^a,b,c,d^
8	−9.42 ± 0.74	−8.15 ± 0.75	−10.24 ± 0.94 ^b^	−15.10 ± 0.72 ^a,b,c^	−22.40 ± 0.90 ^a,b,c,d^
9	−11.20 ± 0.97	−8.43 ± 1.16 ^a^	−10.43 ± 0.64	−14.68 ± 1.16 ^a,b,c^	−21.98 ± 1.54 ^a,b,c,d^
10	−10.48 ± 1.49	−9.41 ± 0.52	−11.88 ± 0.66 ^b^	−17.30 ± 1.65 ^a,b,c^	−22.98 ± 0.78 ^a,b,c,d^
11	−10.76 ± 0.38	−12.84 ± 0.80 ^a^	−13.76 ± 1.41 ^a^	−17.94 ± 0.61 ^a,b,c^	−23.30 ± 1.03 ^a,b,c,d^
12	−13.44 ± 1.08	−14.74 ± 0.82	−17.26 ± 0.70 ^a,b^	−19.34 ± 0.15 ^a,b,c^	−24.92 ± 1.16 ^a,b,c,d^

^a^ Statistically significant differences vs. control group, *p* < 0.05. ^b^ Statistically significant differences vs. modified erythrocyte membranes with PS-NH_2_-200 (C = 0.002 mg/mL), *p* < 0.05. ^c^ Statistically significant differences vs. modified erythrocyte membranes with PS-NH_2_-200 (C = 0.01 mg/mL), *p* < 0.05. ^d^ Statistically significant differences vs. modified erythrocyte membranes with PS-NH_2_-200 (C = 0.1 mg/mL), *p* < 0.05.

## Data Availability

Not applicable.

## Abbreviations

ELS	Electrophoretic light scattering
DLS	Dynamic light scattering
FTIR	Fourier transform infrared
*ζ*	Zeta potential (electrokinetic potential)
PDI	Polydispersity index
PS	Polystyrene particles
PS-100	100 nm polystyrene particles
PS-200	200 nm polystyrene particles
PS-NH_2_-100	100 nm polystyrene particles with amino groups
PS-NH_2_-200	200 nm polystyrene particles with amino groups
RBC	Red blood cells (erythrocytes)
Platelets (thrombocytes)	
